# Supplementary figures and images for: Congenital hearing impairment associated with peripheral cochlear nerve dysmyelination in glycosylation-deficient muscular dystrophy
Source: PLoS Genet. 2020 May 26;16(5):e1008826. doi: 10.1371/journal.pgen.1008826 (PMC7274486; doi:10.1371/journal.pgen.1008826)

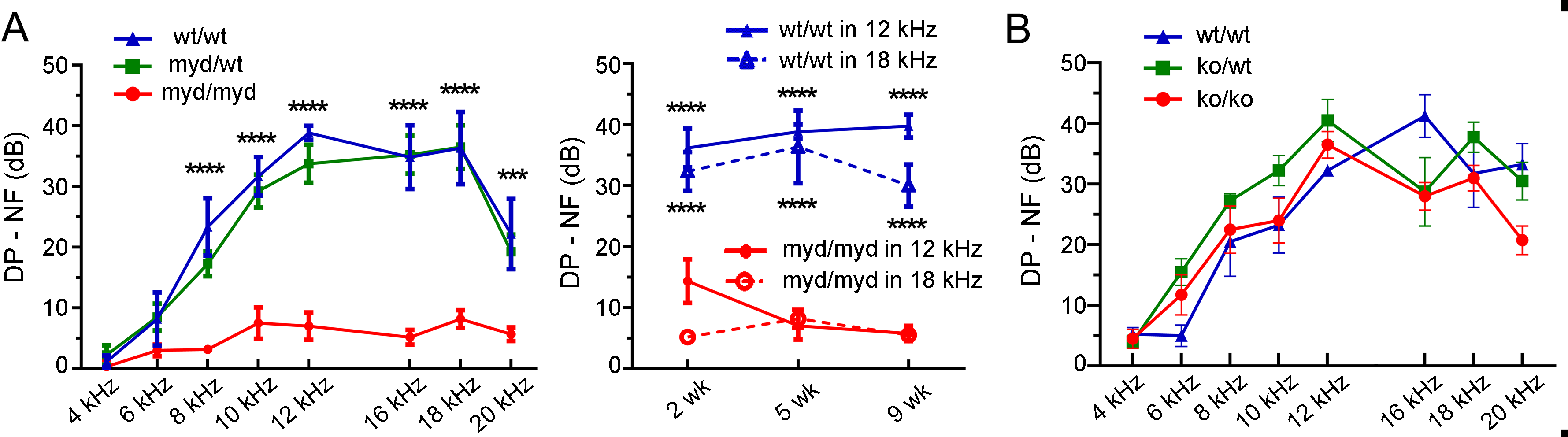

Supplement: S1 Fig — A, DPOAE assessed using pure-tone bursts at 4, 6, 8, 10, 12, 16, 18, and 20 kHz in 5-week-old control (Largewt/wt, n = 6), Largemyd/wt (n = 4), and Largemyd/myd mice (n = 6). DPOAE assessed using pure-tone bursts at 12 and 18 kHz in 2-, 5-, and 9-week-old control mice (n = 6, 6, and 4, respectively) and Largemyd/myd mice (n = 5, 6, and 4, respectively) was graphed. ****P < 0.0001 and ***P = 0.0007 (control vs. Largemyd/myd) using two-way ANOVA with Tukey’s post-hoc test. B, DPOAE assessed with pure-tone bursts at 4, 6, 8, 10, 12, 16, 18, and 20 kHz in 6-week-old control (wt/wt, n = 4), heterozygous POMGnT1-KO (ko/wt, n = 4), and POMGnT1-KO (ko/ko, n = 4) mice. No significant difference was observed. (TIF) [file pgen.1008826.s001.tif]

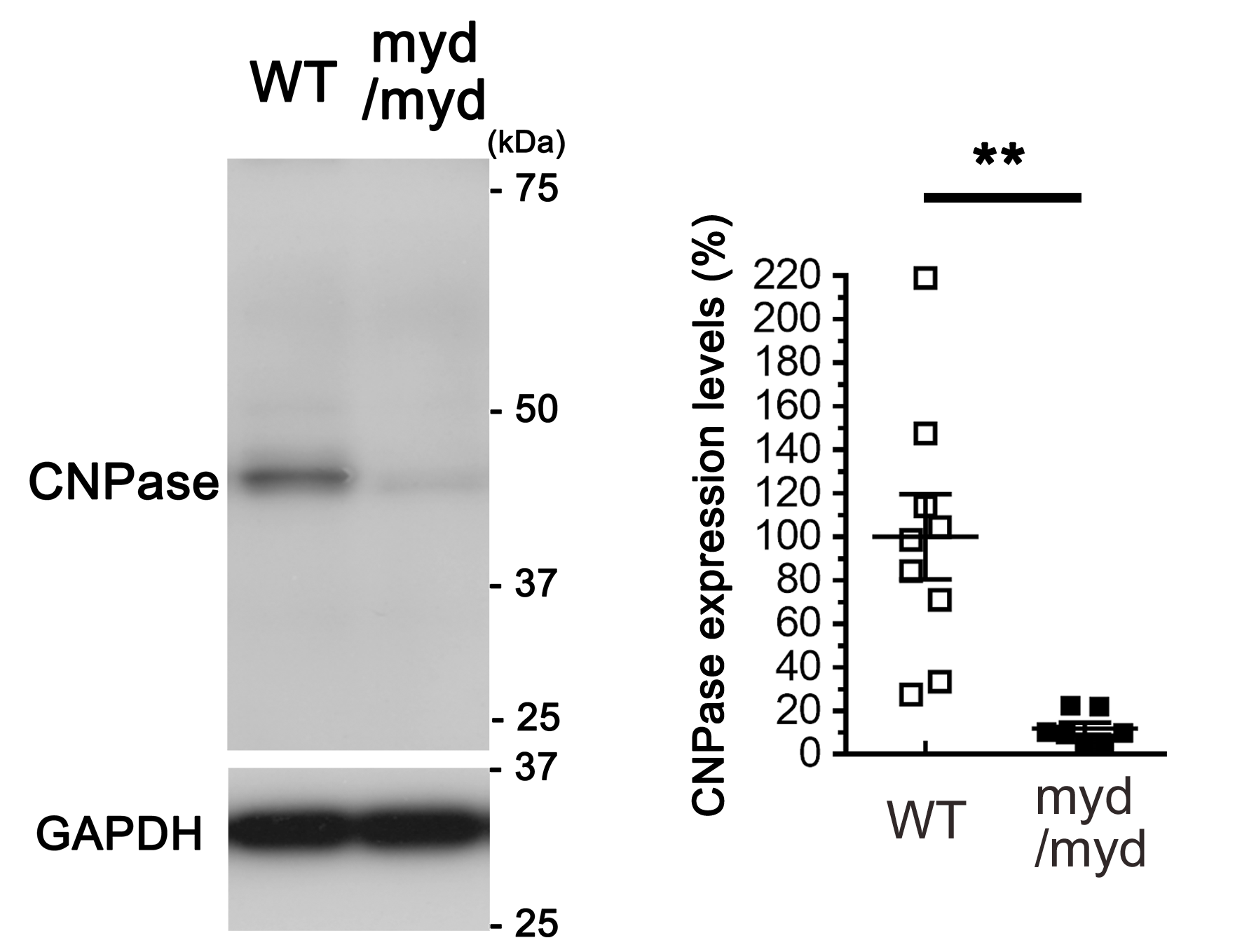

Supplement: S2 Fig — Lysates were obtained from the spiral ganglion (SG)/Rosenthal’s canal (RC) of the P5-7 control and Largemyd/myd mice. CNPase immunoblotting showed decreased levels of CNPase in the Largemyd/myd mice. Comparative loading of proteins was confirmed by immunoblotting of GAPDH. Statistical analysis was performed in pairs of the control (n = 9) and Largemyd/myd mice (n = 7), **P = 0.0015 by Student’s t-test. (TIF) [file pgen.1008826.s002.tif]

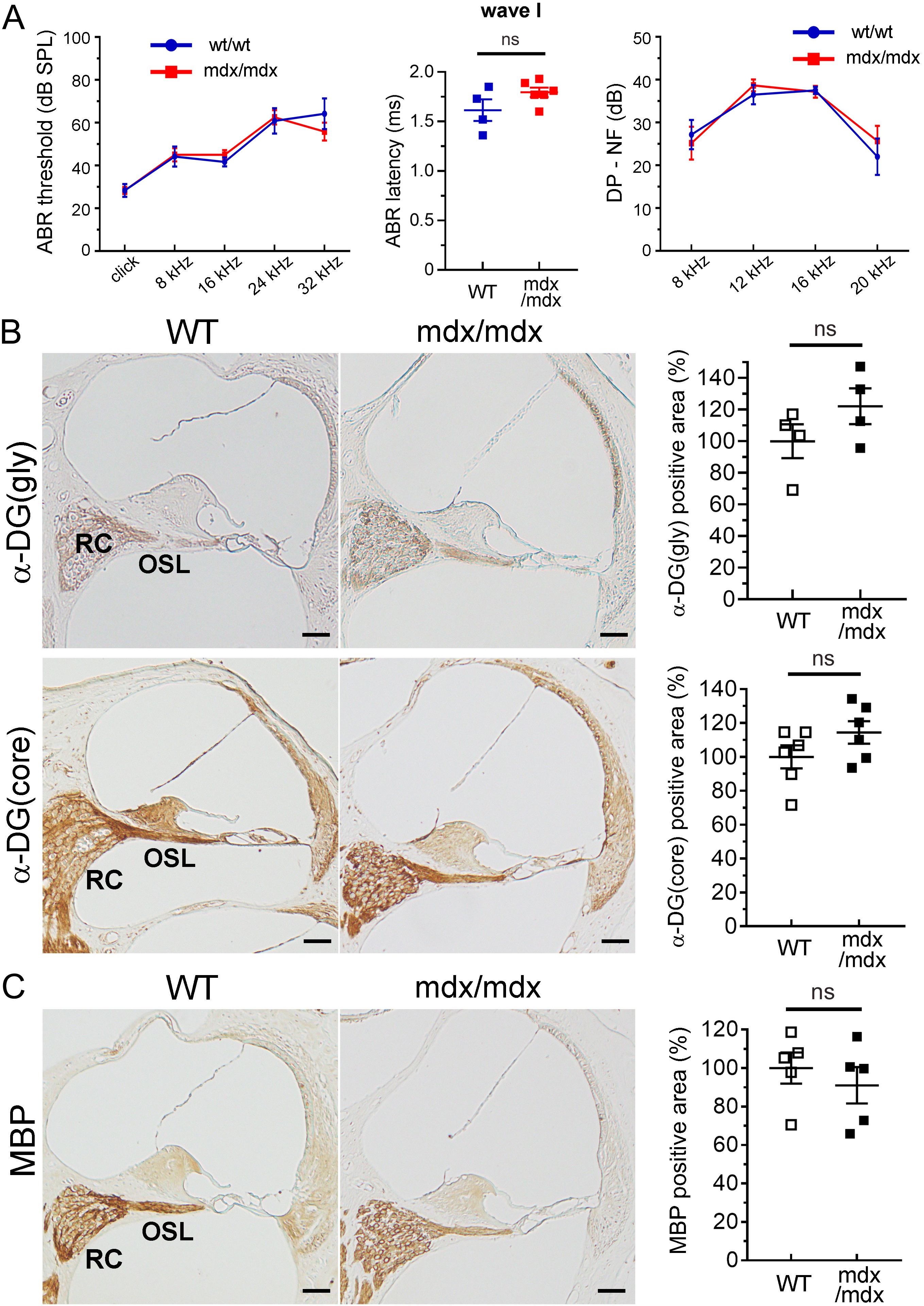

Supplement: S3 Fig — A, ABR with click and pure-tone bursts at 8, 16, 24, and 32 kHz and DPOAE with pure-tone bursts at 8, 12, 16, and 20 kHz were performed in 12-week-old control (Dmdwt/wt, n = 6) and Dmdmdx/mdx mice (n = 6). ABR latency of wave I at the click stimulation of 90 dB in control and Dmdmdx/mdx mice (n = 4 and 6, respectively) were graphed. No significant differences were observed in ABR and DPOAE analyses using two-way ANOVA with Bonferroni's post-hoc test and in ABR latency of wave I analysis using Student’s t-test (P = 0.1230). B and C, Inner ears of 12-week-old control and Dmdmdx/mdx mice were fixed for immunostaining of glycosylated α-DG [α-DG(gly)] and core α-DG [α-DG(core)] proteins (B; n = 4 and 6, respectively) and MBP (C; n = 5). Statistical analysis of immunoreactivity was performed and graphed. No significant difference was observed between control and Dmdmdx/mdx mice using Student’s t-test (P = 0.2039 in α-DG(gly), P = 0.1597 in α-DG(core), and P = 0.4902 in MBP). Scale bars: 50 μm. (TIF) [file pgen.1008826.s003.tif]

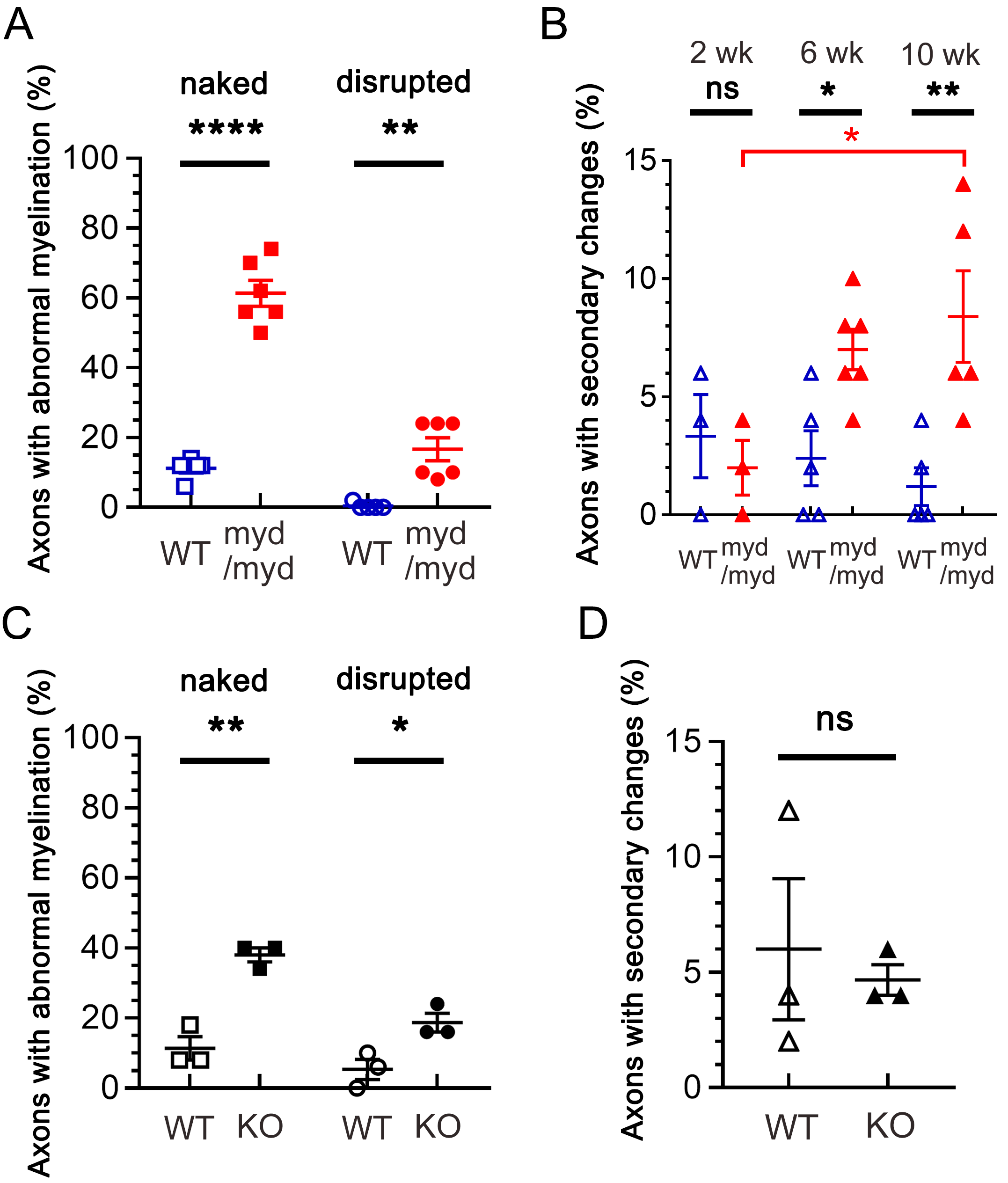

Supplement: S4 Fig — Inner ears of control and Largemyd/myd mice (A, B) and control and POMGnT1-KO mice (C, D) were fixed for transmission electron microscopy (TEM). TEM images at the osseous spiral lamina (OSL) were obtained. Each percentage was obtained by analyzing 50 axons. Statistical analyses were conducted using the Student’s t-test unless indicated. A, Graph showing the percentages of naked axons (****P < 0.0001) and axons with disrupted myelin (**P = 0.0016) in 6-week-old control (n = 5) and Largemyd/myd (n = 6) mice. B, Graph showing the percentage of axons with secondary changes in 2-week-old control and Largemyd/myd mice (n = 3, P = 0.5614), 6-week-old control (n = 5) and Largemyd/myd (n = 6) mice (*P = 0.0100), and 10-week-old control (n = 5) and Largemyd/myd (n = 5) mice (**P = 0.0089). *P = 0.0398 (Largemyd/myd mice at 2 vs. 10 weeks) using one-way ANOVA with Tukey’s post-hoc test. C–D, Graph showing the percentages of naked axons (**P = 0.0024), axons with disrupted myelin (*P = 0.0278), and axons with secondary changes (P = 0.6918) in control and POMGnT1-KO mice (n = 3). (TIF) [file pgen.1008826.s004.tif]

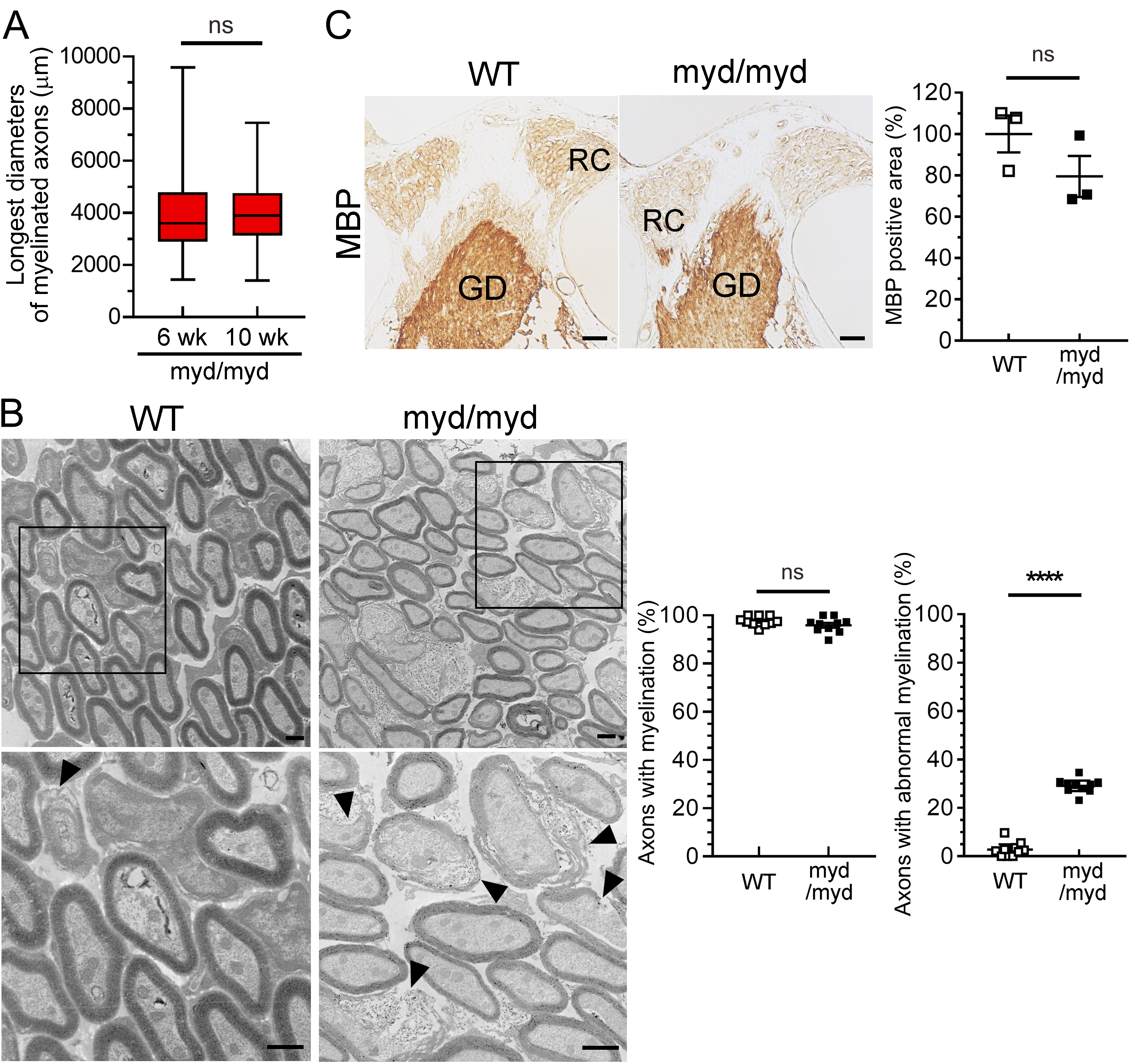

Supplement: S5 Fig — Inner ears of 6- and 10-week-old control and Largemyd/myd mice (A) and 5-week-old control and Largemyd/myd mice (B) were fixed for transmission electron microscopy (TEM). TEM images at the osseous spiral lamina (OSL, A) and proximal to the glial dome (GD) (B, at the region indicated by the asterisk in Fig 4A) were obtained. Cochleae of 8-week-old control and Largemyd/myd mice (C) were fixed for MBP immunostaining. A, The longest diameter of each myelinated axon in the transverse section at the OSL in Largemyd/myd mice at 6 weeks (n = 100) and 10 weeks (n = 74) were statistically analyzed (3 cochleae) using the Kolmogorov–Smirnov test. No significant difference was observed (P = 0.3043). B, The percentage of axons with myelination and of axons with abnormal myelinations in control and Largemyd/myd mice were calculated per x 5000-field, and statistically analyzed (total 10 fields of each obtained from 3 control and 5 Largemyd/myd mice) using the Student’s t-test. Lower panels represent magnified images of the areas indicated by the squares in the upper panels. No significant difference was observed in axons with myelination (P = 0.1657), but a significant difference was observed in axons with abnormal myelination (****P < 0.0001). Arrowheads indicate abnormal myelination. Scale bars: 1 μm. C, Immunostaining was performed using an MBP antibody, and statistical analyses were conducted between control and Largemyd/myd mice (n = 3). No significant difference was observed by Student’s t-test (P = 0.1984). RC: Rosenthal’s canal. Scale bars: 100 μm. (TIF) [file pgen.1008826.s005.tif]

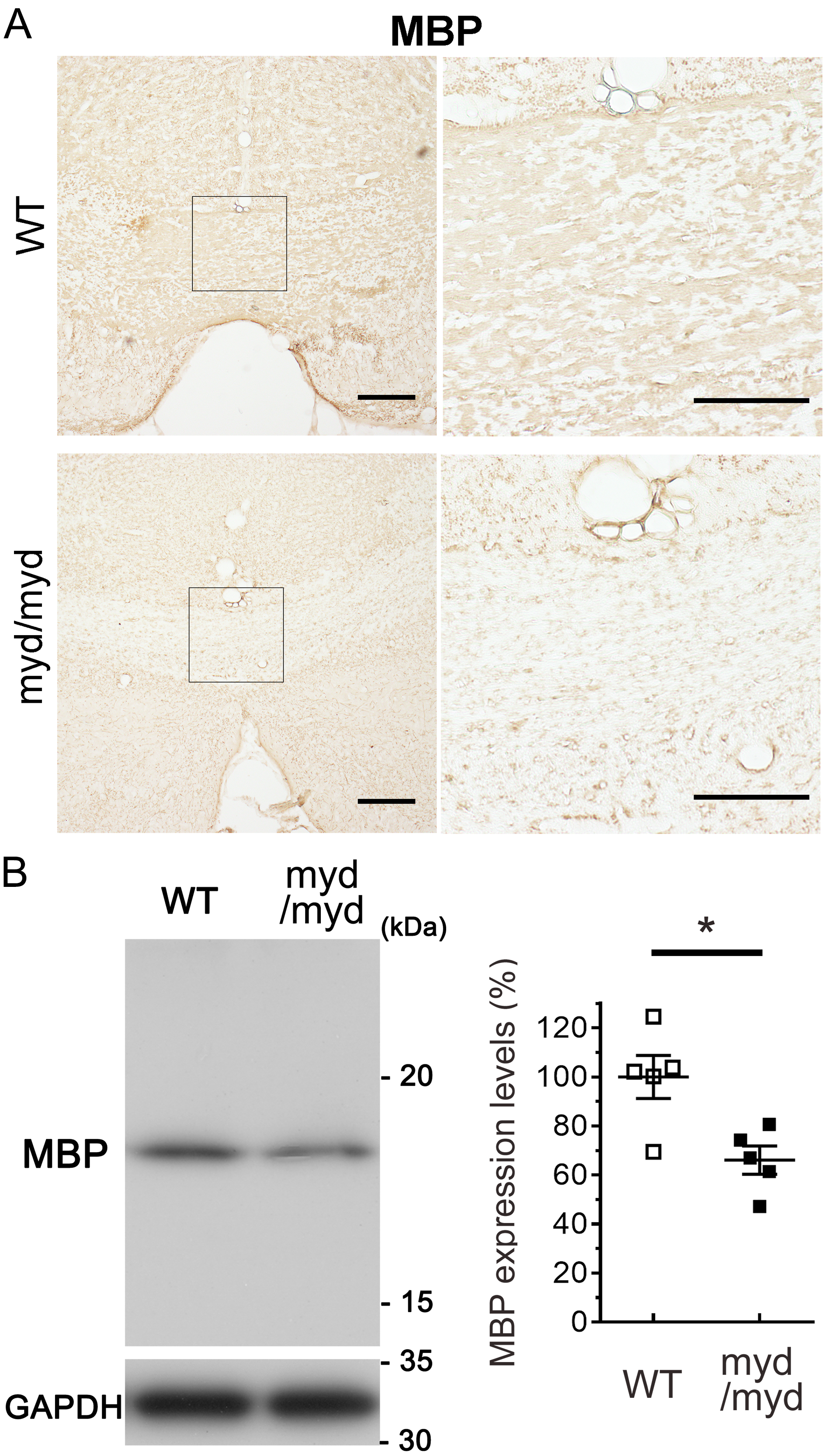

Supplement: S6 Fig — A, Brain sections of eight-week-old control and Largemyd/myd mice at the level of the corpus callosum were obtained for immunostaining for MBP (Scale bars: 200 μm). The panels on the right are magnified images of the corpus callosum indicated by the squares in the panels on the left (Scale bars: 100 μm). Decreased immunoreactivity of MBP was observed in Largemyd/myd mice. The results are presented as the mean of at least three experiments. B, Whole-brain lysates of P7 control and Largemyd/myd mice (n = 5) were obtained for MBP immunoblotting. Decreased expression levels of MBP were observed in Largemyd/myd mice compared with those in control mice. Comparative loading of proteins was confirmed by immunoblotting of GAPDH. *P = 0.0121 by Student’s t-test. (TIF) [file pgen.1008826.s006.tif]

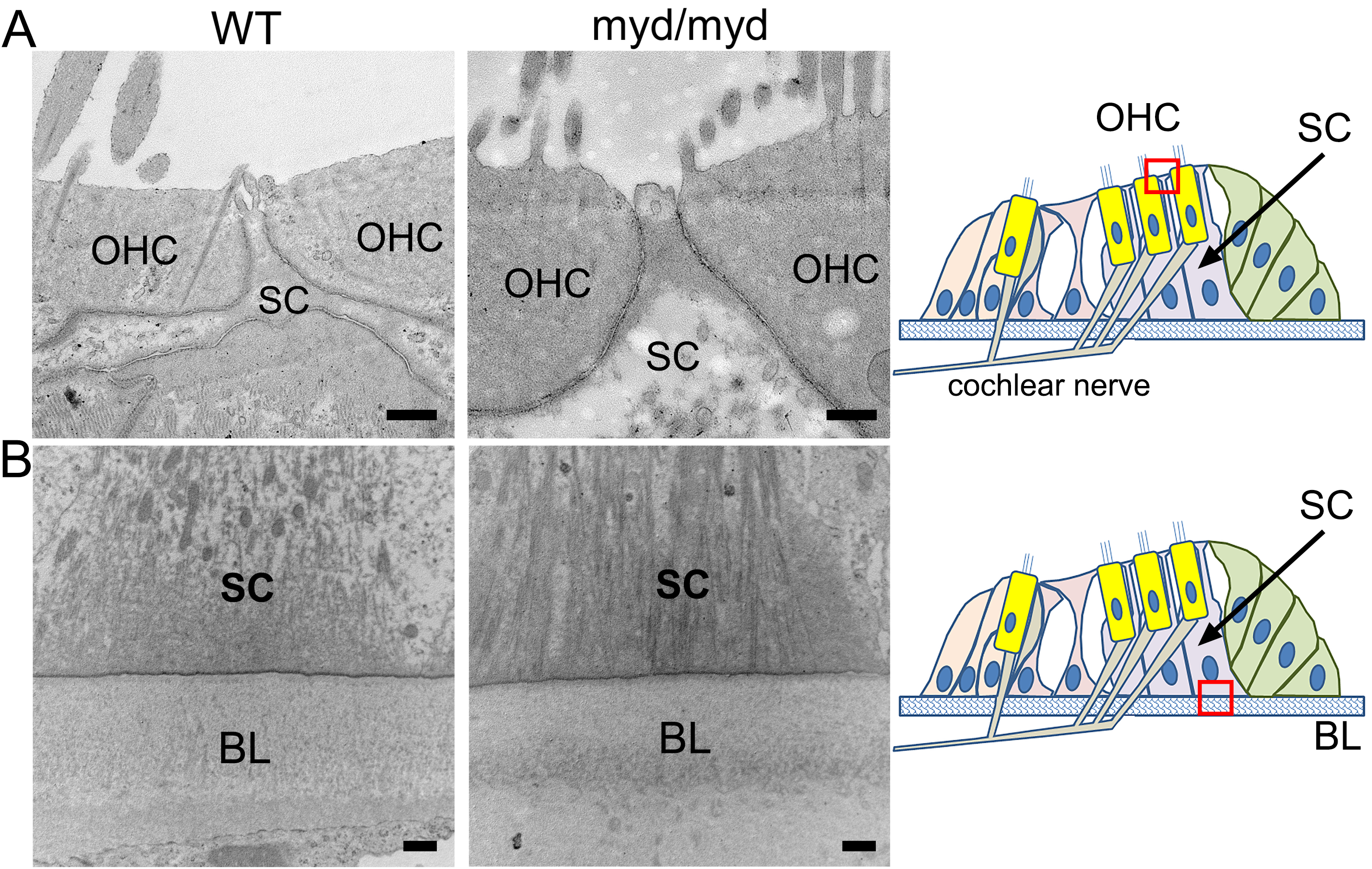

Supplement: S7 Fig — Organs of Corti of 10-week-old controls and Largemyd/myd mice were fixed for transmission electron microscopy (TEM) (A and B). TEM images at the apical connective spaces between outer hair cells (OHCs) and supporting cells (SCs) (A, indicated by the rectangle) and between OHCs and underlying basal lamina (BL) (B, indicated by the rectangle). No apparent difference was observed between control and Largemyd/myd mice. Representative results from three independent experiments are shown (n = 3). Scale bars: 500 nm. (TIF) [file pgen.1008826.s007.tif]
