# Supplementary material for: Congenital hearing impairment associated with peripheral cochlear nerve dysmyelination in glycosylation-deficient muscular dystrophy
Source: PLoS Genet. 2020 May 26;16(5):e1008826. doi: 10.1371/journal.pgen.1008826 (PMC7274486; doi:10.1371/journal.pgen.1008826)
Supplement: S1 Table — Latency of wave I (latency I) and wave V (latency V), interpeak latency between wave I and V (interpeak I-V), and amplitude of wave I (amplitude I) in nine Fukuyama CMD patients analyzed in the present study are shown. Severity is classified based on the physical activity: mild, able to crawl; moderate, able to sit; severe, unable to control head position. CC, cerebellar cyst; ID, intellectual disability. (DOCX) [file pgen.1008826.s008.docx]

**Table S1. ABR analysis of Fukuyama CMD patients.**

| **case** | **genotype** | **months** | **sex** | **Severity** | **involved organs** | **side** | **threshold (dB)** | **latency I (ms)** | **latency V (ms)** | **interpeak**  **I-V (ms)** | **Amplitude I (ms)** |
| --- | --- | --- | --- | --- | --- | --- | --- | --- | --- | --- | --- |
| **1** | SVA Homozygous | 15 | M | Mild | CC, mild ID | R | 40 | 2.32 | 6.67 | 4.35 | 0.04 |
|  |  |  |  |  |  | L | NR | NR | NR | NR | NR |
| **2** | SVA Homozygous | 94 | F | Moderate | CC, mild ID | R | 40 | 1.99 | 5.63 | 3.64 | 0.06 |
|  |  |  |  |  |  | L | 40 | 1.78 | 5.61 | 3.83 | 0.42 |
| **3** | SVA Homozygous | 198 | M | Moderate | CC, moderate ID epilepsy, scoliosis, cardiomyopathy | R | 40 | 2.22 | 6.36 | 4.14 | 0.20 |
|  |  |  |  |  |  | L | 40 | 2.30 | 6.39 | 4.09 | 0.23 |
| **4** | SVA Homozygous | 239 | F | Mild | CC, mild ID | R | 40 | 1.73 | 6.07 | 4.34 | 0.13 |
|  |  |  |  |  |  | L | 40 | 2.12 | 6.18 | 4.06 | 0.09 |
| **5** | SVA/Heterozygous Ex3 | 17 | M | Moderate | CC, moderate ID | R | 40 | 1.88 | 6.07 | 4.19 | 0.09 |
|  |  |  |  |  |  | L | 40 | 2.45 | 7.30 | 4.85 | 0.05 |
| **6** | SVA/Heterozygous Ex3 | 54 | F | Moderate | CC, moderate ID, myopia, dysphagia | R | 40 | 2.82 | 6.31 | 3.49 | 0.15 |
|  |  |  |  |  |  | L | 40 | 2.17 | 5.87 | 3.70 | 0.08 |
| **7** | SVA/Heterozygous Ex3 | 66 | F | Moderate | CC, myopia, moderate ID, myopia | R | 40 | 1.67 | 5.50 | 3.83 | 0.22 |
|  |  |  |  |  |  | L | 40 | 1.73 | 5.74 | 4.01 | 0.08 |
| **8** | SVA/Heterozygous Ex3 | 74 | F | Moderate | CC, myopia, moderate ID, myopia | R | 40 | 1.88 | 6.28 | 4.40 | 0.18 |
|  |  |  |  |  |  | L | 40 | 1.83 | 6.91 | 5.08 | 0.21 |
| **9** | SVA/Heterozygous Int5 | 151 | M | Severe | lissencephaly, CC, retinal detachment, myopia, cataracts, severe ID, cardiac arrest, tracheotomy | R | 40 | 1.73 | 5.94 | 4.21 | 0.08 |
|  |  |  |  |  |  | L | 40 | 1.86 | 6.33 | 4.47 | 0.14 |
|  |  |  |  |  |  |  |  |  |  |  |  |

Latency of wave I (latency I) and wave V (latency V), interpeak latency between wave I and V (interpeak I-V), and amplitude of wave I (amplitude I) in nine Fukuyama CMD patients analyzed in the present study are shown. Severity is classified based on the physical activity: mild, able to crawl; moderate, able to sit; severe, unable to control head position. CC, cerebellar cyst; ID, intellectual disability. NR: no response.
