# Supplementary material for: Congenital hearing impairment associated with peripheral cochlear nerve dysmyelination in glycosylation-deficient muscular dystrophy
Source: PLoS Genet. 2020 May 26;16(5):e1008826. doi: 10.1371/journal.pgen.1008826 (PMC7274486; doi:10.1371/journal.pgen.1008826)
Supplement: S2 Table — Latency of wave I (latency I) and wave V (latency V), interpeak latency between wave I and V (interpeak I-V), and amplitude of wave I (amplitude I) in controls are shown. (DOCX) [file pgen.1008826.s009.docx]

**Table S2. ABR analysis of healthy volunteers (controls) evaluated in the present study.**

| **case** | **months** | **sex** |  | **side** | **threshold**  **(dB)** | **latency I (ms)** | **latency**  **(ms)** | **interpeak I-V (ms)** | **amplitude I**  **(ms)** |
| --- | --- | --- | --- | --- | --- | --- | --- | --- | --- |
| **1** | 15 | M |  | R | 40 | 1.67 | 5.74 | 5.55 | 0.19 |
|  |  |  |  | L | 40 | 1.70 | 5.84 | 5.52 | 0.32 |
| **2** | 17 | F |  | R | 40 | 1.59 | 5.74 | 5.70 | 0.04 |
|  |  |  |  | L | 40 | 1.52 | 5.63 | 5.58 | 0.05 |
| **3** | 53 | M |  | R | 40 | 2.06 | 5.79 | 5.39 | 0.40 |
|  |  |  |  | L | 40 | 1.67 | 5.74 | 5.57 | 0.17 |
| **4** | 60 | F |  | R | 40 | 1.70 | 6.67 | 6.56 | 0.11 |
|  |  |  |  | L | 40 | 1.54 | 6.46 | 6.40 | 0.06 |
| **5** | 66 | M |  | R | 40 | 1.59 | 5.63 | 5.59 | 0.04 |
|  |  |  |  | L | 40 | 1.65 | 5.55 | 5.18 | 0.37 |
| **6** | 90 | F |  | R | 40 | 1.36 | 6.12 | 6.10 | 0.02 |
|  |  |  |  | L | 40 | 1.36 | 5.94 | 5.90 | 0.04 |
| **7** | 141 | F |  | R | 40 | 1.67 | 5.61 | 5.40 | 0.21 |
|  |  |  |  | L | 40 | 1.65 | 5.66 | 5.52 | 0.14 |
| **8** | 206 | F |  | R | 40 | 1.54 | 6.13 | 6.11 | 0.02 |
|  |  |  |  | L | 40 | 1.65 | 5.71 | 5.65 | 0.06 |
| **9** | 293 | M |  | R | 40 | 1.54 | 5.61 | 5.48 | 0.13 |
|  |  |  |  | L | 40 | 1.73 | 5.79 | 5.67 | 0.12 |

Latency of wave I (latency I) and wave V (latency V), interpeak latency between wave I and V (interpeak I-V), and amplitude of wave I (amplitude I) in controls are shown.
