# Supplementary material for: Congenital hearing impairment associated with peripheral cochlear nerve dysmyelination in glycosylation-deficient muscular dystrophy
Source: PLoS Genet. 2020 May 26;16(5):e1008826. doi: 10.1371/journal.pgen.1008826 (PMC7274486; doi:10.1371/journal.pgen.1008826)
Supplement: S3 Table — Participant number, sex, mean age, and ABR data were compared between all Fukuyama CMD patients (total) and controls. (DOCX) [file pgen.1008826.s010.docx]

**Table S3. Comparison between all Fukuyama CMD patients and controls.**

|  | Fukuyama CMD  (total) | control |  |
| --- | --- | --- | --- |
| number | 9 | 9 |  |
| sex (male/female) | 4/5 | 4/5 |  |
| mean age (months) | 104.6 | 105.9 |  |
| hearing threshold < 40dB (ears) | 17 | 18 |  |
| wave I latency (ms) | 2.03 ± 0.32 | 1.62 ± 0.15 | * |
| wave I amplitude (μV) | 0.14 ± 0.12 | 0.14 ± 0.09 |  |
| wave V latency (ms) | 6.19 ± 0.48 | 5.85 ± 0.31 | * |
| wave V amplitude (μV) | 0.38 ± 0.24 | 0.26 ± 0.12 |  |
| interpeak latency I-V (ms) | 4.16 ± 0.41 | 4.23 ± 0.36 |  |
|  |  |  | * *P* < 0.05 |

Participant number, sex, mean age, and ABR data were compared between all Fukuyama CMD patients (total) and controls.
