# Supplementary material for: Congenital hearing impairment associated with peripheral cochlear nerve dysmyelination in glycosylation-deficient muscular dystrophy
Source: PLoS Genet. 2020 May 26;16(5):e1008826. doi: 10.1371/journal.pgen.1008826 (PMC7274486; doi:10.1371/journal.pgen.1008826)
Supplement: S4 Table — Participant number, sex, mean age, and ABR data were compared between Fukuyama CMD patients with homozygous (homo) mutations and controls. (DOCX) [file pgen.1008826.s011.docx]

**Table S4. Comparison between Fukuyama CMD patients with homozygous mutations and**

**controls.**

|  | Fukuyama CMD (homo) | control |  |
| --- | --- | --- | --- |
| number | 4 | 4 |  |
| sex (male/female) | 2/2 | 2/2 |  |
| mean age (months) | 153.9 | 151.0 |  |
| hearing threshold < 40dB (ears) | 7 | 8 |  |
| wave I latency (ms) | 2.07 ± 0.24 | 1.57 ± 0.15 | * |
| wave I amplitude (μV) | 0.17 ± 0.13 | 0.11 ± 0.10 |  |
| wave V latency (ms) | 6.13 ± 0.40 | 5.86 ± 0.19 | * |
| wave V amplitude (μV) | 0.31 ± 0.09 | 0.30 ± 0.14 |  |
| interpeak latency I-V (ms) | 4.06 ± 0.26 | 4.29 ± 0.30 |  |
|  |  |  | * *P* < 0.05 |

Participant number, sex, mean age, and ABR data were compared between Fukuyama CMD patients with homozygous (homo) mutations and controls.
