# Supplementary material for: Congenital hearing impairment associated with peripheral cochlear nerve dysmyelination in glycosylation-deficient muscular dystrophy
Source: PLoS Genet. 2020 May 26;16(5):e1008826. doi: 10.1371/journal.pgen.1008826 (PMC7274486; doi:10.1371/journal.pgen.1008826)
Supplement: S5 Table — Participant number, sex, mean age, and ABR data were compared between Fukuyama CMD patients with heterozygous (hetero) mutations and controls. (DOCX) [file pgen.1008826.s012.docx]

**Table S5. Comparison between Fukuyama CMD patients with heterozygous mutations and controls.**

|  | Fukuyama CMD (hetero) | control |  |
| --- | --- | --- | --- |
| number | 5 | 5 |  |
| sex (male/female) | 2/3 | 2/3 |  |
| mean age (months) | 72.4 | 67.4 |  |
| hearing threshold < 40dB (ears) | 10 | 10 |  |
| wave I latency (ms) | 2.00 ± 0.37 | 1.66 ± 0.15 | * |
| wave I amplitude (μV) | 0.13 ± 0.06 | 0.16 ± 0.13 |  |
| wave V latency (ms) | 6.23 ± 0.54 | 5.85 ± 0.39 | * |
| wave V amplitude (μV) | 0.22 ± 0.14 | 0.44 ± 0.28 |  |
| interpeak latency I-V (ms) | 4.22 ± 0.50 | 4.18 ± 0.42 |  |
|  |  |  | * *P* < 0.05 |

Participant number, sex, mean age, and ABR data were compared between Fukuyama CMD patients with heterozygous (hetero) mutations and controls.
