# Supplementary material for: Congenital hearing impairment associated with peripheral cochlear nerve dysmyelination in glycosylation-deficient muscular dystrophy
Source: PLoS Genet. 2020 May 26;16(5):e1008826. doi: 10.1371/journal.pgen.1008826 (PMC7274486; doi:10.1371/journal.pgen.1008826)
Supplement: Attachment — Submitted filename: Step-by-step response_PLOS Genetics_after editing_200227.doc [file pgen.1008826.s021.doc]

Morioka et al., “**Congenital hearing impairment associated with peripheral cochlear nerve dysmyelination in glycosylation-deficient muscular dystrophy**”, manuscript ID PGENETICS-D-19-01933.

We gratefully thank the reviewers for their careful review and constructive advice for revision of our manuscript. In accordance with the reviewers’ advice, we have performed certain new experiments, carefully reviewed previous literature, and rewritten the manuscript by adding new data and references. These revisions are highlighted in yellow and blue in the manuscript.

During revision of the manuscript, we found two additional mistakes that we would like the editor and reviewers to be aware of in fairness of publishing our results. They are as follows:

1) Fig. 2B (wave I amplitude): The statistical analysis that was performed using n = 3~4, is now performed using n = 5~6, as shown in the legend and the raw data. The new analysis has the same significant difference, as shown in the original legend (**P = 0.0014 changed to ****P > 0.0001).

2) Fig. 3B: The y-axis had the wrong graph scale, it was divided into four starting from 0 and finishing at 2.0, however, it should have started from 1.0 and finished at 2.0. We have corrected the error in the graph. This change has not influenced the statistical analysis, which has remained the same (*P = 0.0197).

Additionally, in accordance with submission guidelines, we have uploaded the raw data Figures 2-7 and Figures S1-S6 (those of Figure 8 are shown Table S1 and S2).

**Author responses to the Editor’s and Reviewers’ comments**

**Reviewer #1**

*1. The title is not accurate. The studies convincingly show that alpha-dystroglycan and laminin protein levels are decreased at the peripheral cochlear nerve and that the mice do not hear well, but the experiments do not directly link these two observations. In fact, it is striking that the hearing phenotypes differ in the two mouse models: DPOAEs are essentially absent in the myd/myd mice but normal in the POMGnT1-KO mice. Since there is a strong DPOAE phenotype in the myd/myd mice, it is impossible to interpret the decreased wave 1 amplitude and increased latency, as these changes could be secondary to abnormal cochlear function*. *The POMGnT1-KO mice show only a transient change in wave I latency though thresholds are elevated at all ages. Since the abnormal myelination is shown at 10 weeks in that mutant, a time when latencies are recovered, it seems like the change in myelination cannot be responsible for the change in latency. Also, since both mice show changes in alpha-dystroglycan glycosylation and dysmyelination in the peripheral cochlear nerve yet only one strain shows a DPOAE phenotype, these cellular phenotypes are unlikely to be the full explanation for the observed hearing loss. Other interpretations need to be considered.*

**Response**:

We thank the reviewer for the thoughtful comments. In accordance with the reviewer’s comment, we have changed the title of our manuscript to “Congenital hearing impairment associated with peripheral cochlear nerve dysmyelination in glycosylation-deficient muscular dystrophy.”

We agree with the reviewer that our study is weak regarding the direct linkage between cellular/histological phenotypes and the hearing phenotype: myd/myd mice show decreased/absent DPOAE, and *POMGnT1*-KO mice show improved latency of wave I in ABR at 10 weeks of age. Muscular dystrophy-dystroglycanopathy (MD-DG) is a complex disease, which causes many lesions in many organs/tissues including the peripheral nerve, brainstem, and brain, suggesting that the hearing impairment is also caused by complex mechanisms. As described in the original manuscript, cortico-olivocochlear efferent pathways may cause the decreased/absent OAE (Jager et al.; cited as ref. 44). In the revised manuscript, we have discussed cortico-olivocochlear efferent pathways in more detail and with additional references (Danesh et al., Lopez-Poveda et al., Guinan et al.; newly cited as ref. 45-47). Charcot-Marie-Tooth (CMT) disease types 1A and 1B, which are congenital neuropathies caused by abnormal myelination by Schwann cells, sometimes show decreased/absent DPOAE (Kovach et al., newly cited ref. 76, regarding CMT1A; Starr et al., newly cited ref. 49, regarding CMT1B). Indeed, mild morphological changes (atrophy) were reported at the stria vascularis, where endocochlear potential is generated, in CMT1B (Starr et al., Nadol et al.; newly cited as ref. 49-50). Additionally, myotonic dystrophy type 1 (DM1), which is a type of MD frequently presenting with hearing impairment, shows abnormal OAE (Pisani et al., Balatsouras et al.; cited as ref. 18-19), suggesting that there may be an as yet unknown but common cellular mechanism between DM1 and MD-DG resulting in OHC dysfunction. Although the mechanism of decreased/absent DPOAE in *myd*/myd mice is still unclear, we have described these possibilities in the revised Discussion section with additional supporting references (lines 280-296, page 14).

Regarding the recovered wave I latency in ABR observed in *POMGnT1*-KO mice, *POMGnT1*-KO mice have fewer secondary changes in axons themselves than *myd/myd* mice do (we have added data showing that 10-week-old *POMGnT1*-KO mice had no significant increase in the number of axons with secondary changes compared with controls [S4D Fig], in contrast to that in *myd/myd* mice [S4B Fig]). In addition, *POMGnT1*-KO mice analyzed in the present study are those that survived (less than 40% of all births). Therefore, judged on the basis of morphological phenotypes, *POMGnT1*-KO mice in the present study may have a much greater potential for functional recovery than *myd/myd* mice with secondary changes in the axons. Alternatively, in addition to the lesion at the peripheral segment of the cochlear nerve, *POMGnT1*-KO mice may have other lesions which affect the early hearing phenotype but improve by 10 weeks of age. We have described and discussed the improved hearing phenotype in *POMGnT1*-KO mice in the revised Discussion section (lines 306-312, page 15) and added new supporting data (S4B and S4D Fig).

*2. The authors demonstrate changes in alpha-dystroglycan and laminin using both immunohistochemistry and Western blot. However, it is hard for me to interpret the immunohistochemistry since it is an enzymatic detection using DAB substrate, which is not a quantitatively reliable assay. The authors should note whether the control and mutant tissues were processed on the same slides and developed for the same amount of time. This information was not provided in the Methods section.*

**Response:**

We thank the reviewer for the careful assessment of our manuscript. We performed the immunohistochemistry using DAB with great attention for quantitative analysis, which is supported by the immunoblotting analysis with regard to key aspects. We compared sample pairs (control vs. mutant) prepared (fixed, embedded into paraffin, cut into slices, and immunostained) on the same day and as per the same schedule and conditions, such as the same duration of developing times. To improve and increase the reliability of our immunohistochemistry data and to ensure that our methods can be replicated if needed, we have added this information to the revised Methods section (lines 455-457, page 21).

*3. The dysmyelination phenotype is striking but needs to be better quantified. I do not understand what is meant by “dispersion of the longest diameter in the myelinated axon” (p. 10, 212-210). This made it very hard for me to assess the data shown in Fig. 6. Please explain exactly what was measured and how. Likewise, I do not understand the definition of “normal myelinated axons” or how that was assessed objectively. Since the effect on myelination in both mouse lines is a key finding, these data should be better quantified overall. For instance, Fig 6 illustrates naked axons, disrupted myelin, vacuolated axons, and aggregated axons. The authors should quantify the incidence of each of these pathologies, blind to genotype, in mutants and littermate controls.*

**Response:**

We thank the reviewer for the constructive comments and advice. In accordance with the reviewer’s comment, we have described what we mean by “the longest diameter in the myelinated axon” and how this was measured and quantified in the revised Methods section (lines 526-529, page 24). To clearly show this in Fig. 6C (now new Fig. 7C), we have renamed the y-axis “Number of myelinated axons.” In addition, we have modified the description “dispersion of the longest diameter in the myelinated axon at the OSL was broader” to “diameters of myelinated axons in the transverse section at the OSL was more broadly distributed” (lines 210-211, page 10).

As the reviewer suggested, defining “normal myelinated axons” or “axons with normal myelination” is not easy. Hence, we decided to show abnormal myelination or abnormal axons. We have categorized abnormal axons into three categories: “naked axons”, “axons with disrupted myelin”, and “axons with secondary changes (vacuoles and/or aggregates)”. “Axons with abnormal myelination” was defined as the sum of “naked axons” and “axons with disrupted myelin.” We have described this definition and the quantitative method in the revised Method section (lines 522-526, page 24). In accordance with the definition and modification, we have modified the graphs in Figs. 7B (2-, 6-, and 10-week-old *myd/myd* mice) and 7E (10-week-old *POMGnT1*-KO mice), and the right graph of S5B Fig (*myd/myd* mice), which now show the percentage of axons with abnormal myelination. Additionally, we have analyzed and quantified the “naked axons” and “axons with disrupted myelin” in 6-week-old myd/myd mice and sibling controls (new S4A), and “axons with secondary changes” in 2-, 6-, and 10-week-old *myd/myd* mice and sibling controls (new S4B Fig), and “naked axons”, “axons with disrupted myelin”, and “axons with secondary changes” in 10-week-old *POMGnT1*-KO mice and sibling controls (new S4C and S4D Fig). Accordingly, we have made the relevant changes to the revised Result sections (lines 203-223, page 10).

With the re-analysis and addition of samples based on the new definitions, an additional statistically significant difference (*P = 0.0399) was observed in the percentage of axons with abnormal myelination between 2-week-old and 6-week-old *myd/myd* mice (Fig. 7B). This seems to be a result of separately analyzing “axons with abnormal myelination” and “axons with secondary changes” in the new analysis, whereas both were grouped together in the original analysis. Indeed, the number of “axons with secondary changes” in myd/myd mice was significantly increased in 6-, but not 2-week-old mice. Accordingly, we have made the relevant changes to the revised Result sections (lines 204-210, page 24).

*4. The authors note that “None of the FCMD patients analyzed had complaint of deafness” (line 236, page 11), but go on to show that wave 1 latencies were increased. The authors should discuss this point in the Discussion, as it has an impact on the clinical value of the information presented. For instance, please discuss how increased latencies might affect hearing and whether there are assays that might reveal a deficit that is otherwise missed by both the audiologists and the patients.*

**Response:**

We thank the reviewer for the thoughtful suggestion. Regarding the increased latency of wave I in ABR, several studies have suggested that prolonged latency and reduced amplitude of wave I are characteristic findings for tinnitus patients with normal hearing (Milloy et al.; newly cited as ref. 70). Recently, “hidden hearing loss” has been referred to as hearing dysfunction not detected by standard tests of auditory thresholds, but can be diagnosed by a reduction of ABR wave I amplitude in the absence of ABR threshold or latency changes (Kohrman et al.; newly cited as ref. 71). In addition to cochlear synaptopathy, which is induced by impaired synaptic connection between hair cells and the cochlear nerve (Liberman et al.; newly cited as ref. 72), peripheral neuropathy including demyelination of the cochlear nerve has been reported as a cause of hidden hearing loss (Kohrman et al.; ref. 71). Demyelination-related hidden hearing loss reportedly had the additional phenotype of prolonged (and permanent) wave I latency in ABR in Guillain-Barre syndrome (Wan et al.; newly cited as ref. 73). Additionally, demyelination-related hidden hearing loss with abnormal speech recognition ability in noisy backgrounds, but normal ABR amplitude and latency, was reported in CMT1A (Choi et al.; newly cited as ref. 74). Thus, decreased speech understanding and altered temporal processing in noisy environments despite the normal threshold in standard audiometric tests are likely diagnosed as “hidden hearing loss”, which may involve or be associated even with tinnitus and hyperacusis (Choi et al., Rance et al., Liberman et al.; newly cited as ref. 74, 75, and 78). We described the clinical value of the prolonged latency of ABR wave I and its importance and potential methods for its detection (speech recognition test in noisy backgrounds and estimation of auditory temporal resolution/processing) in the revised Discussion section (lines 322-335, page 15-16 & lines 347-354, page 16-17).

*5. Since the primary cellular phenotype seems to be a change in myelination, there should also be some discussion of how the hearing phenotype compares to what is observed in other mouse models with impaired myelination.*

**Response:**

We thank the reviewer for the constructive comment. The most famous congenital neuropathies caused by impaired myelination accompanied by hearing phenotypes are CMT1A and CMT1B, which are the most and second common types of CMT, respectively. Sensorineural hearing loss first manifesting in adolescence is a relatively common phenotype in CMT1, but it is highly variable. CMT1A and CMT1B are caused due to genetic abnormalities in peripheral myelin protein 22 and myelin protein zero, which are the main myelination-associated proteins in Schwann cells. To our knowledge, no study has focused on hearing function and pathology of the cochlear nerve using mouse models of CMT1A or CMT1B; however, a few studies have focused on hearing phenotypes and their pathology in humans. Although Kovach et al. (newly cited as ref. 76) regarding CMT1A and Starr et al. (newly cited as ref. 49) regarding CMT1B reported abnormal ABR accompanied with decreased/absent OAE, the principal pathology of CMT1B is the myelin damage of the cochlear nerve initiating distally, accompanied by secondary damage to the spiral ganglion neurons itself, without loss of inner hair cells (Starr et al., Nadol et al.; newly cited as ref. 49-50). While children with CMT1A showed no prolonged latency of wave I (Rance et al.; ref. 75), adult patients showed prolonged latency of wave I (Giuliani et al.; newly cited as ref. 77). Demyelination-related hidden hearing loss reportedly had the additional phenotype of permanently increased wave I latency in ABR in Guillain-Barre syndrome (Wan et al.; newly cited as ref. 73). Additionally, hidden hearing loss with abnormal speech recognition in noisy backgrounds, but normal ABR amplitude and latency, was reported in CMT1A (Choi et al.; newly cited as ref. 74). Thus, both CMT1 and MD-DG probably have the principal lesion at the distal portion of the cochlear nerve; however, CMT1 shows a progressive phenotype (Choi et al., and Giuliani et al., newly cited ref. 74 and 77). These differences are likely to arise from the different etiologies of CMT1 and MD-DG (autosomal dominant vs. autosomal recessive inheritance, respectively), and the degrees of cochlear synaptopathy involvement may also be different in the two diseases. We described these arguments in the revised Discussion section (lines 336-354, page 16-17) combined with our response regarding the comment 4 (lines 322-335, page 15-16 & lines 347-354, page 16-17).

*6. For the patient analysis, the authors should report auditory thresholds so that the effect on latencies can be properly interpreted. I am also curious whether patients with“mild”motor phenotypes also had“mild”effects on latency.*

**Response:**

We thank the reviewer for the thoughtful comment. In addition to the overall analysis of Fukuyama CMD patients, we also analyzed them by dividing into two groups: patients with homozygous SVA mutations and those with compound heterozygous mutations. Generally, Fukuyama CMD phenotypes are more severe in patients with compound heterozygous than those with homozygous SVA mutations (Ishigaki et al., Kondo-Iida et al.; cited as ref. 10 and 73) (Table S1); we have added this description to the revised Methods section (lines 402-404, page 19). Judging from our data analyzed by genotype (homo vs. hetero), there are no significant differences in wave I latency of ABR (Fig. 8B and C, S4 and S5 Tables; 2.07 ± 0.24 ms vs. 2.00 ±0.37 ms). For example, (see S1 Table; case 1 [homo with mild MD, 15 months] vs. case 5 [hetero with moderate MD, 17 months]), there are no apparent difference in wave I latency (2.32 ms (right)/no response (left) vs. 1.89 ms /2.45 ms). Contrarily, for case 4 (homo with mild MD, 239 months) vs. case 3 (homo with moderate MD, 198 months), there seems to be a difference with regard to severity of MD phenotype (1.73 ms/2.12 ms vs. 2.22 ms/2.30 ms. Additionally, only one patient with severe MD phenotype in the hetero group (case 9, 151 months) had a shorter wave I latency (1.73 ms/1.86 ms) than the average of the group (2.00 ± 0.37 ms). These results suggest that severity of MD phenotype is not correlated with the phenotype on wave I latency in ABR. Because our analysis was limited to nine Fukuyama CMD patients in the present study, we believe that further studies with larger number of FCMD patients, and hopefully focusing on both Fukuyama CMD and other types of MD-DG, will be required to clarify the relationship between severity of MD phenotype and hearing impairment, including analysis of wave I latency in ABR. We have described our results—no relation between severity of MD phenotypes and the hearing phenotype (prolonged wave I latency) in Fukuyama CMD—in the revised Discussion section (lines 389-392, page 18).

*Minor points:*

*1. I expect this paper will be interesting to hearing researchers who are unlikely to be familiar with the various forms of muscular dystrophy. The introduction needs to be improved to make it easier for non-experts to follow the differences between the three mouse models presented as well as the human form of MD that is assessed. This could be achieved by reworking the introduction and perhaps adding a figure introducing the various enzymes related to the mouse and human mutants that are ultimately examined.*

*2. I do not think it is appropriate to use a phrase such as “To prove our hypothesis.” This wording suggests that the authors wanted to be right. It is more appropriate to say “To test our hypothesis.” This may seem minor but I think it is important to demonstrate a value for unbiased and objective approaches in official scientific publications.*

*3. The paper should be proofread and edited for improved clarity. For instance, I believe “torn bursts” is meant to be “tone bursts” (line 420, p. 20) and “spinal ganglion” should be “spiral ganglion” (line 822, p. 36).*

**Response:**

1. Thank you for the constructive suggestion. In accordance with the reviewer’s suggestion, we have added an illustration (as new Figure 1) to better explain MD-DG (by showing the disease types and corresponding defective enzymes). We believe that the illustration greatly improves the readability and understanding of the Introduction.

2. In accordance with the reviewer’s suggestion, we have changed “To prove our hypothesis” to “To test our hypothesis”.

3. Thank you for the careful reading of our manuscript. We have proofread our manuscript again and corrected the errors as follows: “torn bursts” to “tone bursts” and “spinal ganglion” to “spiral ganglion.” Furthermore, our manuscript was re-edited by an experienced editor.

**Reviewer #2**

*1. One complaint is that the title of the manuscript suggests the authors have discovered a causal link between the loss of alpha-dystroglycan and hearing loss. This is not at all accomplished and so a more conservative title is strongly recommended.*

**Response:**

We thank the reviewer for the thoughtful comment. In accordance with the reviewer’s comment, we have changed the title to a more conservative one as follows: “**Congenital hearing impairment associated with peripheral cochlear nerve dysmyelination in glycosylation-deficient muscular dystrophy**.”

*2. The Discussion should provide more information on important future directions,*

*1) like an investigation of the temporal and spatial expression patterns of Large and Pomgnt1.*

*2) There should also be a more thorough discussion of possible defects in efferent fibers and OHC function, which could underlie the DPOAE defects reported.*

*3) Also, have the Schwann cells started to die off at some point? Are they poorly differentiated? These questions should also be addressed in the Discussion.*

**Response:**

We thank the reviewer for the thoughtful and constructive comments. In accordance with the reviewer’s suggestions, we have modified several sections in the Discussion.

1) Previous reports focusing on the temporal and spatial expression patterns of Large and POMGnT1 are limited. Using the limited references (Peyrard et al., Fujimura et al., Yoshida et al.; newly cited as ref. 33-35), we have added descriptions about the temporal and spatial expression patterns of Large and POMGnT1. We have added this information in the revised Discussion section (lines 260-265, page 13).

2) Regarding the effect of cortico-olivocochlear efferent fibers to OHCs on DPOAE, we have added new information and cited additional references (Danesh et al., Lopez-Poveda et al., Guinan et al.; newly cited as ref. 45-47) useful for future studies and readers. Although the discrepancy of DPOAE data between *myd/myd* and *POMGnT1*-KO mice still remains unexplained, we have described and discussed this issue in the revised Discussion section (lines 280-296, page 14).

3) According to previous reports, the laminin-dystroglycan complex is reportedly more important for the myelination/differentiation of Schwann cells (Berti et al.; newly cited as ref. 43) as well as oligodendrocytes (Colognato et al., Development, 2007) rather than for their survival/proliferation. We have described this information in the revised Discussion section (lines 273-274, page 13).

*A few minor issues:*

*1. The authors use excessive acronyms and these add a lot of visual clutter to the narrative. I suggest choosing only two or three terms to reduce to acronyms, and to otherwise spell out everything else.*

*2. The statement in the last two sentences of the 2nd paragraph (Discussion section, line 278-281) needs references to back up the hypothesis.*

*3. Figure 4D and S2B: Can authors show quantitative data for this experiment?*

*4. Line 399: Is this reference to figure 4C accurate?*

*5. Figure S6: Is this figure missing quantitative data? The Figure legend says that the results are presented as mean of at least 3 representative experiments.*

*6. Figure S6: The cartoon has some thick lines extending from HCs. What are these?*

**Response:**

We thank the reviewer for the constructive advice and comments.

1. In accordance with the reviewer’s suggestion, we have reduced the number of acronyms as much as possible, while minimizing the total word count of the manuscript. We have not used acronyms for dystrophin–dystroglycan complex, Duchenne MD, Becker MD, dystroglycan (except in case of α-dystroglycan and β-dystroglycan), basal lamina, *FUKUTIN, fukutin related protein,* α-dystroglycanopathy, Fukuyama CMD, antibody, white matter lesions, and paraformaldehyde. In addition, to increase readability, we have not used α-DG for α-dystroglycan in the Abstract and Author Summary. These changes are highlighted in blue.

2. In accordance with the reviewer’s comment, we have modified the speculation [more mildly explained with a new reference 48-50 (Ishiyama et al., Starr et al., and Nadol et al.); lines 289-294, page 14] and added a new speculation (lines 292-296, page 14) with references to support the statements made.

3. In accordance with the reviewer’s suggestion, we planned to perform additional experiments using *POMGnT1*-KO mice during the times we had. However, because *POMGnT1*-KO mice have a high mortality around the period of genotyping procedure, we were unable to obtain the sufficient number (at least 3) of *POMGnT1*-KO mice needed for statistical analysis. Hence, we have presented scatterplots of Fig. 4D and S2B Fig (now, new Fig. 5D and new S2C Fig, respectively) in S2A Fig (n; 1 in WT, 2 in Hetero, and 2 in KO) and S2C Fig (n; 2 in WT, 1 in Hetero, and 2 in KO) without *P* values, respectively.

4. We have corrected the error by changing Fig. 4C into Fig. 3C (now, new Fig. 4C).

5. We examined the ultrastructure of the organ of Corti in myd/myd mice using at least three littermate pairs (WT vs. *myd/myd* mice); however, we were unable to observe morphological abnormalities. It is impossible to quantitatively show negative data; hence, we have presented the representative image showing no abnormality/difference between *myd/myd* and control mice. To more clearly indicate this result, we have modified the legend as follows:

“The representative results from 3 independent experiments are shown (n= 3).”

6. We intended to show nerves attaching to HCs, but the image was confusing. For better clarity, we have modified the cartoon and added a caption explaining the thick lines (cochlear nerve).
