# Supplementary material for: Congenital hearing impairment associated with peripheral cochlear nerve dysmyelination in glycosylation-deficient muscular dystrophy
Source: PLoS Genet. 2020 May 26;16(5):e1008826. doi: 10.1371/journal.pgen.1008826 (PMC7274486; doi:10.1371/journal.pgen.1008826)
Supplement: Attachment — Submitted filename: Step-by-step response_PLOS Genetics_Re-revision_200430final.doc [file pgen.1008826.s022.doc]

Morioka et al., “**Congenital hearing impairment associated with peripheral cochlear nerve dysmyelination in glycosylation-deficient muscular dystrophy**”, manuscript ID PGENETICS-D-19-01933R2.

The authors would like to thank the reviewer for his/her constructive critique to improve the manuscript. We made every effort to address the issues raised and to respond to all comments. The answers are highlighted in yellow in the manuscript (marked-up copy). In accordance with the reviewer’s advice, we have revised the Result section, Figure S2, and the corresponding legend.

**Author responses to the Editor’s and Reviewer’s comments**

**Reviewer #2**

The authors put forth great effort to address the concerns that came from the first submission. The manuscript is well-suited for publication. One note -- during the revisions, the authors apparently had some survival issues in their POMGnT1-KO mice, which left them with limited sample numbers for quantifying Western blots. They included what they had in Supplementary Figures 2A and 2C. However, the small sample size precluded statistical significance and so statistical data are not shown. Given the small sample size, my opinion is that these data are preliminary and not suitable for publication. I suggest the authors remove Supplementary Figures 2A and C and note the trends and limited sample numbers in the Results section.

**Response:**

We would like to thank the reviewer for the constructive comment and suggestion. In accordance with the reviewer’s comment, we have removed Figure S2A and S2C, and have changed the description regarding these results in the Results section (lines 180-182 and line 191, page 9) and in the legend of Figure S2. In addition, we have added a description regarding Figure 5D (lines 180-182, page 9), which shows the trends of the decreased pan-laminin levels in the *POMGnT1-KO* mice compared with the controls, obtained from a limited sample number.
